# An Unusual Presentation of Diaphragmatic Rupture

**DOI:** 10.7759/cureus.51001

**Published:** 2023-12-23

**Authors:** Malaz S Younis, Alya E Abdelmageed, Khaled Aljohani, Mohammad Alsenani

**Affiliations:** 1 Trauma Surgery, King Saud Medical City, Riyadh, SAU; 2 Trauma and Acute Care Surgery, King Saud Medical City, Riyadh, SAU

**Keywords:** rupture of the spleen, shattered spleen, abdominal trauma, diaphragmatic rupture, diaphragmatic herniation, diaphragmatic injuries

## Abstract

Representing less than 1% of all traumatic injuries, diaphragmatic injuries are uncommon and are usually associated with injuries in other thoracic and abdominal organs.

We report a case of a diaphragmatic injury in a 38-year-old man who presented to the Emergency Department due to a pedestrian-vehicle accident. He had a massive hemothorax on the left due to a ruptured spleen. An exploratory laparotomy was done to manage the bleeding, restore the diaphragmatic hernia contents in their right anatomical position, conduct a splenectomy, and repair the diaphragmatic defect.

Although the majority of diaphragmatic ruptures are diagnosed acutely, late presentations are usually reported in blunt trauma; therefore, a high clinical suspicion with imaging is essential for the diagnosis.

## Introduction

Diaphragmatic rupture following trauma is a rare condition occurring in 0.46% of all abdominal traumatic injuries [1], with young males in their thirties being the most vulnerable group. In a study which reviewed 1000 cases of traumatic diaphragmatic rupture, it was found that 685 (68.5%) of the injuries were left-sided, 242 (24.2%) right-sided, 15 (1.5%) bilateral, and 9 (0.9%) pericardial ruptures; 49 cases were unclassified [2]. Other related injuries such as rib fractures and renal injuries were seen in about 50% of the patients [3].

Diaphragmatic injuries can occur in multiple ways: directly via a penetrating thoracoabdominal trauma or indirectly via severe blunt trauma, causing a sudden increase in intraabdominal pressure.

In a review of the National Trauma Data Bank, of those with diaphragmatic injury, 33% had a blunt mechanism, and 67% had a penetrating mechanism [4]. In addition, up to 90% of blunt diaphragm ruptures were due to motor vehicle accidents, while the remainder were caused by falls or crush injuries [5].

Here, we report an unusual case of Grade V splenic injury (by means of blunt trauma) that was initially assumed as a massive haemothorax. Radiological imaging revealed an actively bleeding shattered spleen in the left thoracic cavity due to a diaphragmatic rupture, along with other intra-abdominal organs.

## Case presentation

A 38-year-old patient was received by our Emergency Department following a pedestrian accident where he was entrapped between two vehicles, resulting in thoracoabdominal trauma. Upon admission, the patient exhibited full alertness and orientation, as indicated by a Glasgow Coma Scale score of 15/15. Vital signs were documented, showing a heart rate of 110 beats per minute, blood pressure measuring 110/60 mmHg, a respiratory rate of 28 breaths per minute, and oxygen saturation of 94% on 15 liters of oxygen via face mask. The patient's initial complaint was pain in the left hypochondrium and lower lumbar regions. Subsequently, an extended-focused assessment with sonography (eFAST) was performed, revealing the presence of free fluid in the splenorenal area. No gross hematuria was detected.

**Figure 1:**
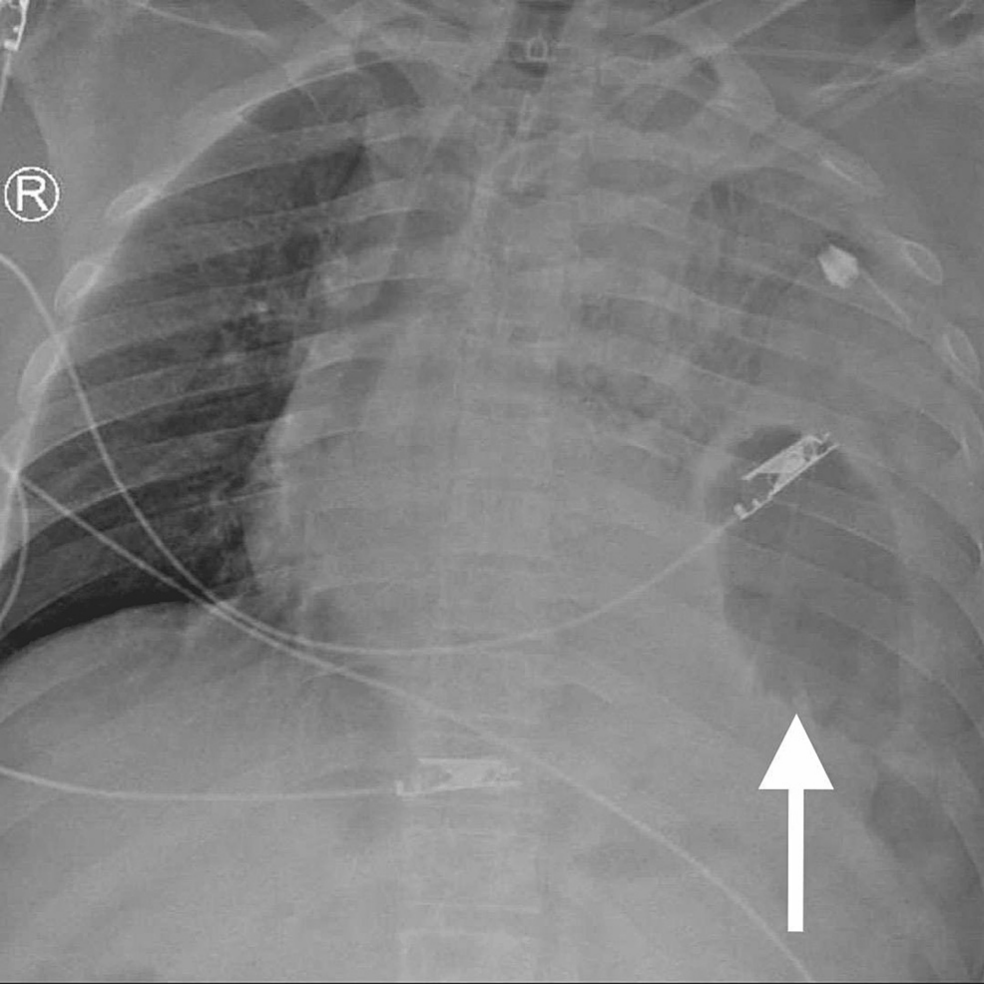
Chest X-ray showing a mediastinal shift towards the right with multiple left rib fractures, and opacities in the left thorax.

In Figure 1, the initial chest X-ray indicated a deviation of the mediastinum toward the right side, the presence of multiple left rib fractures, and the existence of heterogeneous opacities in the upper left lung region.

**Figure 2:**
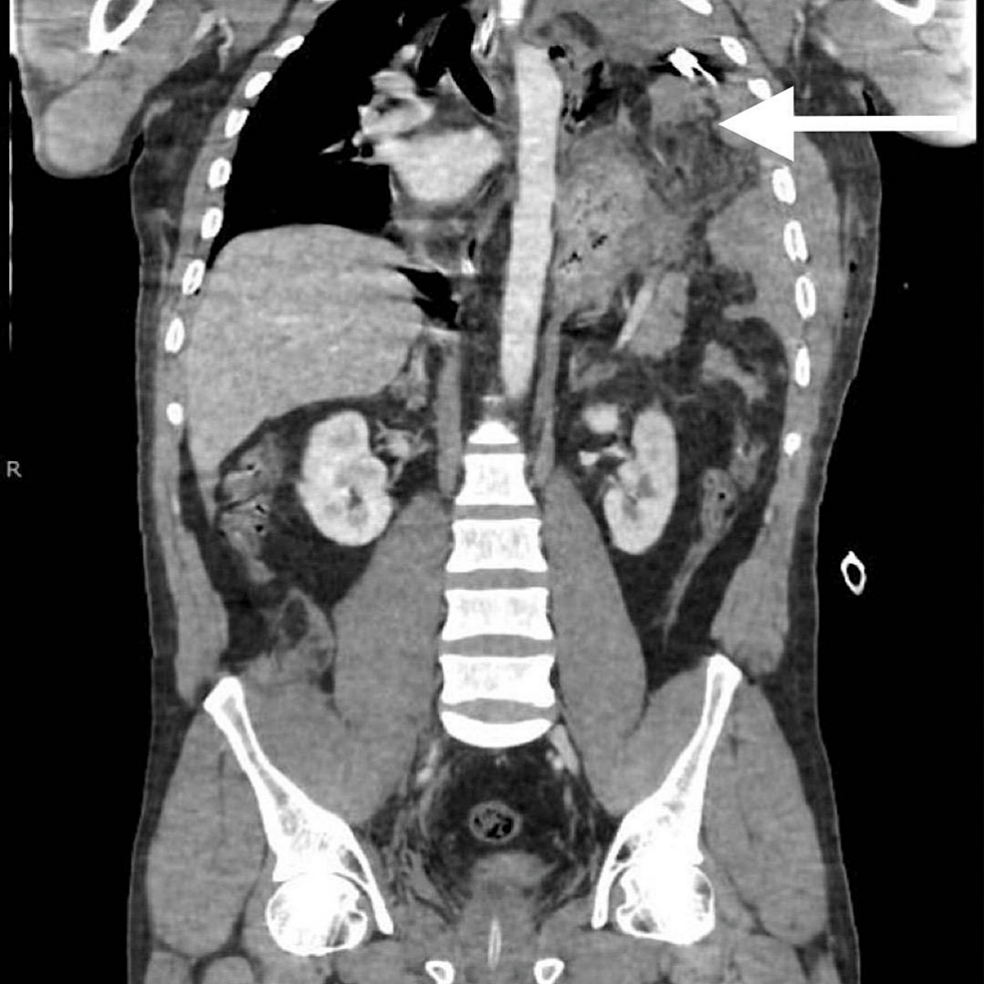
CT scan (coronal view) showing abdominal visceral herniation (arrow) into the left hemithorax.

Subsequently, the patient underwent a CT scan (as depicted in Figure 2), unveiling the presence of a significant left diaphragmatic hernia that enclosed the stomach, spleen, and tail of the pancreas. The hernia imposed substantial pressure, leading to a mass effect affecting the left lung and mediastinum. Furthermore, the CT scan detected a partial collapse of the left lung with lobar airspace opacities, as well as the displacement of the mediastinum towards the right side, which culminated in the development of a left-sided hemopneumothorax.

The spleen displayed a Grade V laceration, indicative of a shattered spleen, with active extravasation stemming from the splenic artery, with a concern of avulsion. Additionally, the left kidney exhibited a Grade II injury, characterized by several areas of disrupted parenchyma extending to the hilum, with no signs of ongoing extravasation. Furthermore, a minor hematoma was identified along the inferior pole of the liver, manifesting as a subtle linear hyperdensity measuring approximately 17 mm in hepatic segment two, likely representing a Grade 1 injury. Lastly, a fracture of the right transverse processes of the L1-L3 vertebrae was observed, along with a pre-vertebral hematoma and displaced fractures of the 7th, 8th, and 9th left ribs.

An exploratory laparotomy was performed, revealing a 12 cm defect in the posterior region of the diaphragm. Within the left pleural cavity, various organs, including the stomach, omentum, transverse colon, splenic flexure, and the entire spleen, were found. A Grade V splenic injury, associated with active bleeding, was also observed, and the chest cavity contained approximately one liter of blood mixed with clots, along with multiple fragments of splenic tissue.

**Figure 3:**
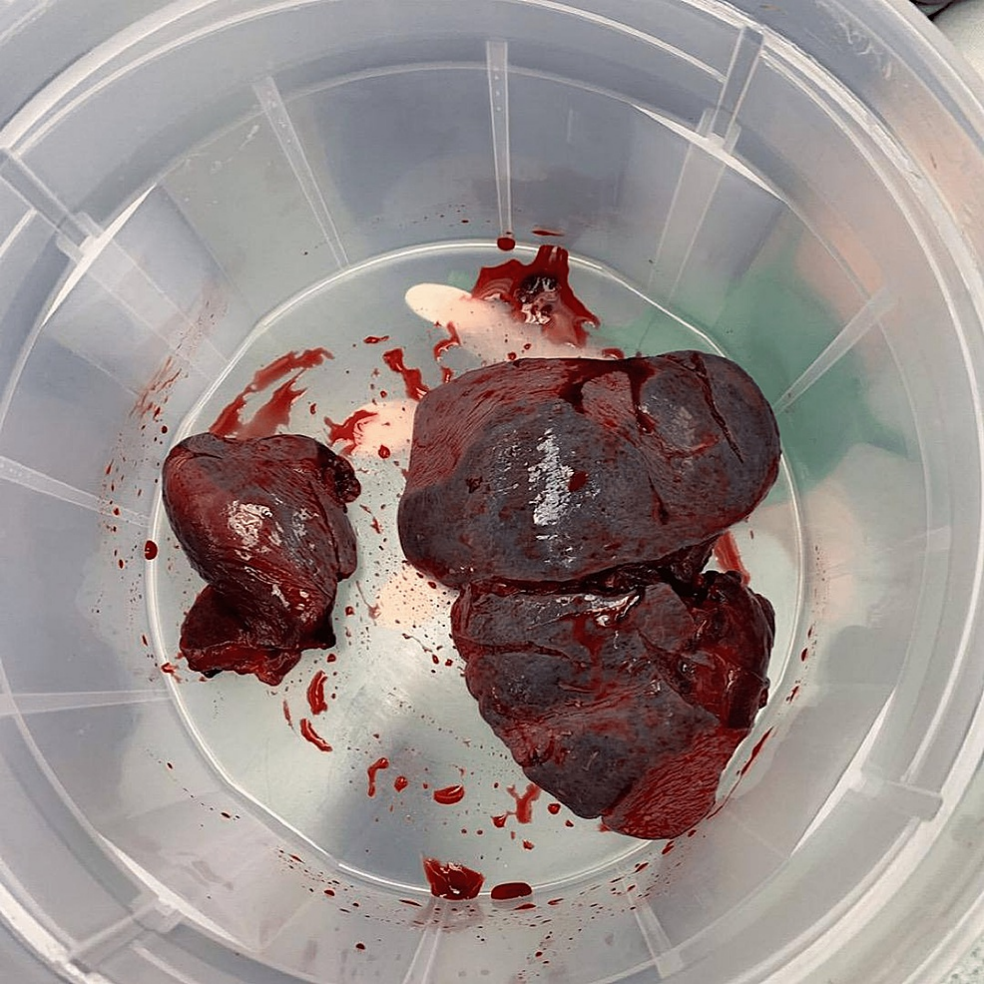
Shattered spleen post-splenectomy (placed in a bucket).

The diaphragmatic defect was then enlarged to reduce the herniated contents, and a splenectomy was conducted (Figure 3). The left pleural cavity was then washed thoroughly through the diaphragm. Following this, the defect was closed via simple repair, and the diaphragm was repaired using Prolene sutures (Ethicon-Johnson & Johnson, USA).

**Figure 4:**
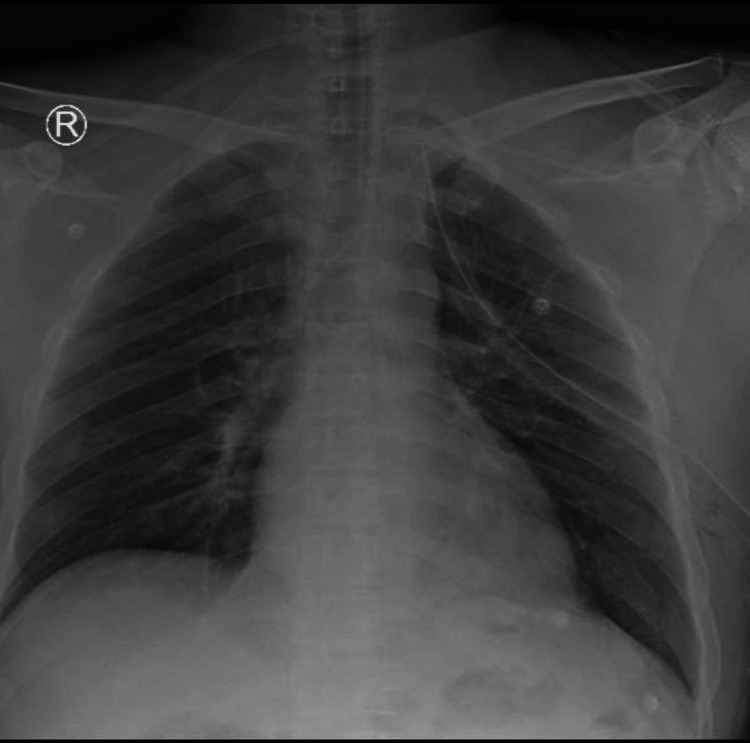
Chest X-ray after the operation with a chest tube in place.

The exploration of the lesser sac, pancreas, and bowel yielded no noteworthy findings. A chest tube was inserted, as shown in Figure 4, and a pelvic drain was placed.

The patient's hospitalization period proceeded without any significant events, and his renal parameters, initially recorded as urea 7.6, serum creatinine 171.6, and potassium, remained within the normal range of 3.79 mmol throughout his stay. Regular daily assessments of renal function tests indicated a consistent downward trend until the readings fell within the normal range.

The pelvic drain was removed after four days, and the chest tube was removed after five days. Given the patient's post-splenectomy status, he received *Haemophilus influenza* and pneumococcal conjugate vaccines, with plans to administer the meningococcal vaccine four weeks later. Ultimately, the patient was discharged on the 10th day following the surgery.

## Discussion

While diaphragmatic injuries can sometimes present with evident signs, such as the herniation of abdominal contents visible on chest radiographs, the diagnosis of traumatic diaphragmatic rupture is frequently delayed due to the absence of distinctive signs and symptoms, as well as inconclusive initial imaging studies. Consequently, maintaining a high index of suspicion is crucial, as a delayed diagnosis can lead to life-threatening complications.

In cases where the diagnosis is uncertain, healthcare providers may employ diagnostic laparoscopy, thoracoscopy, or open surgical exploration to establish a definitive diagnosis [6]. The most commonly observed clinical symptoms of diaphragmatic rupture include marked respiratory distress and diffuse abdominal pain [7]. Swift diagnosis in such situations can be achieved at the patient's bedside through the use of eFAST. This approach can reveal the presence of fluid collection in the subphrenic and pleural spaces or detect small bowel mucosal folds in instances of bowel herniation. Additionally, portable X-rays can be utilized, and the diagnosis can be reinforced with additional imaging techniques such as CT scans [8].

The most important features in chest X-rays that suggest diaphragmatic rupture include the loss of diaphragmatic contour, diaphragm elevation (more pronounced on the left side), the absence of a gastric bubble, mediastinal shift, and the presence of pneumothorax, hemothorax, and hemopneumothorax [9]. However, it is important to note that the overall accuracy of chest X-rays in diagnosing diaphragmatic injuries is relatively low. Sensitivity has been reported to range from 27% to 62% for left-sided injuries and 18% to 33% for right-sided injuries [10].

CT scans serve as valuable and essential tools for detecting diaphragmatic herniation resulting from blunt trauma. Critical signs include the direct visualization of the injury, herniation of visceral contents into the thoracic cavity, and active contrast extravasation. These findings collectively indicate a diaphragmatic injury [11]. Furthermore, multi-detector CT scans have demonstrated a sensitivity of 78% and specificity of 100% in identifying left-sided diaphragmatic injuries [12]. Although MRI may have a role in diagnosing such cases, no substantial studies have examined its accuracy thus far.

Notably, a similar case has been reported in Nepal by Dahal et al., where the spleen and stomach herniated into the left hemithorax following blunt abdominal trauma from a motor vehicle accident [13]. Another case reported by Konstantino et al. in Greece in which the left kidney and the entire spleen were found herniated into the left chest, completely disconnected from their vascular pedicle and vascular tree, respectively, due to blunt trauma. Chest X-ray revealed significant left hemothorax without any additional indications of diaphragmatic injury. Subsequent imaging was not performed and the patient was promptly taken to surgery [14].

## Conclusions

Although diaphragmatic rupture is rare, it requires a high index of suspicion regardless of the mechanism of injury. If the CT abdomen result is equivocal, serial chest X-ray studies or diagnostic laparoscopy would be helpful in detecting diaphragmatic injury early. Additionally, enlarging small diaphragmatic defects is useful to reduce herniated viscera safely, examine the pleural cavity manually, and remove all retained blood and clots.
